# Emotional labor and absenteeism among early childhood educators: The mediating roles of negative affect and psychological meaningfulness

**DOI:** 10.1016/j.heliyon.2024.e40039

**Published:** 2024-11-01

**Authors:** Seth Yeboah Ntim, Collins Opoku Antwi, Michael Osei Aboagye, Elijah Takyi Mensah, Emmanuel Tetteh Teye, Xinyu Li

**Affiliations:** a School of Psychology, Zhejiang Normal University, Jinhua, 321004, China; b Center for Tourism Studies, College of Geography & Environmental Science, Zhejiang Normal University, Jinhua, 321004, China; c Department of Interdisciplinary Studies, Akenten Appiah-Menka University of Skills Training and Entrepreneurial Development, Kumasi AK-039, Ghana; d International Education School, Guangzhou College of Technology and Business, Guangzhou, Guangdong, China; e Department of Adult and Higher Education, Montana State University, USA

**Keywords:** Emotional labor, Negative affect, Psychological meaningfulness, Absenteeism, Early childhood education, Preschool teachers

## Abstract

Teacher absenteeism is one of the key factors that has been fingered as the bane of quality early childhood education in low- and middle-income countries. Failing to report to school as scheduled is considered symptomatic of emotional dysregulation. However, limited research has explored emotional labor as a possible predictor of teacher absenteeism. Therefore, this study, using the conservation of resources theory, examines the influence of emotional labor (i.e., surface and deep acting) on absenteeism, and the mediating roles of negative affect and psychological meaningfulness. Our study used cross-sectional data from 574 preschool teachers in Ghana and structural equation modeling (SEM) to test the hypotheses. The results reveal that surface acting increases absenteeism. Further, negative affect and psychological meaningfulness partially mediated surface acting and absenteeism relationship, but psychological meaningfulness fully mediated deep acting and absenteeism relationship. This study supports the theoretical assumption that teacher absence from school is a resource-based process that is associated with surface acting directly and indirectly via negative affect and psychological meaningfulness. Theoretical and practical implications of our findings are discussed.

## Highlights

• This study examined emotional labor, negative affect, psychological meaningfulness, and teacher absenteeism relationships.
• Surface acting predicted absenteeism.
• Negative affect and psychological meaningfulness mediated surface acting and absenteeism relationship.
• Psychological meaningfulness mediated deep acting and absenteeism relationship.

## 1 Introduction

The governments in low- and middle-income countries have committed to making sure that no child is left behind by expanding early childhood education [1]. Because it is through early education that children learn social and emotional skills and awareness such as turn-taking, teamwork, and following instructions [2]. However, such expansion interventions have occurred without adequate investment in quality [3], causing disparities in early childhood education within [4] and across low- and middle-income countries [5]. Eagle et al. [3] estimated 6.4 %–17 % benefit-to-cost ratio (i.e., the benefits society gain compared to the cost incurred) for increasing enrolment from about 25 % to 50 % in the context of low- and middle-income countries [3]. For example, Ghana recorded about 75 % net enrollment; however, high enrollment does not equate effective learning, resulting in “a very shallow learning profile” [6]. This is very worrying. That children just go through school without education; the very purpose for which they go.

Research has identified several factors that derail quality education in low- and middle-income countries. Particularly in Ghana, Wolf and Colleagues found teacher professional development [[9], [10], [11], [12]] and household socioeconomic status and parental investment [7,8] as key to quality early childhood education. Aside training and parents’ economic situation, other scholars (e.g., Ref. [13]) have emphasized motivation crisis among preschool teachers in Ghana. Another principal factor that has been highlighted in literature but given little attention is teacher absenteeism [14,15].

Referred to as the failure to report to school as scheduled [16], teacher absenteeism is a pervasive problem in low- and middle-income countries [14]. In one Indian government primary school, an unexpected visit by supervisors found 25 % of the teachers to be absent [17]. In Sri Lanka, teacher absenteeism accounted for 15 % of total working school days [18]. To put these figures in perspective, Guerrero et al. [19], through a systematic review, found that teachers in low- and middle-income countries absent themselves 3 %–27 % in a school year. The effect of teacher absenteeism on students’ socio-emotional learning and academic performance is well documented [15,18]. However, little is known about the factors that contribute to teacher absenteeism in low- and middle-income countries. Initial attempts implicate workplace distal factors including high student-teacher ratio, low professional development as well as infrequent and meager remuneration [13,20]. But limited attention has been given to the proximal psychosocial outcomes of working in such an environment that may give rise to absenteeism.

We argue that the high student-teacher ratio paired with minimal teacher training in Ghana may create an emotional demand situation that undermines preschool teachers’ emotional management and self-presentation. For instance, a teacher handling a class of 40 or more students, scenarios like the following occur everyday: s/he has to calm two or more crying kids with others fighting or wanting to use the bathroom. Such situations may lead the teacher to adopt maladaptive emotion regulation strategy. That is, such a teacher may be emotionally at his or her wits end but may keep a cheerful demeanor as expected of professional teachers. Managing felt and expressed emotions to achieve organizational goals is termed as emotional labor [21]. Preschool teachers may labor emotionally by modifying felt emotions (i.e., deep acting) or suppressing felt emotion (i.e., surface acting) to create a nurturing and supportive environment for young learners [[21], [22], [23]].

In teaching and learning, teachers who deep act experience more positive affect and positive job outcomes such as positive attitude towards work, job satisfaction, and commitment [24]. However, teachers who engage in surface acting experience strain. Strain drains psychological, emotional, and physiological resources, leading to ill-being and negative work attitude and behavior (e.g., burnout, attrition, lateness, and presenteeism) [25]. Although these studies have offered rich theoretical and empirical support for emotional labor and employees’ outcomes, scant studies exist on the long-lasting manifestations of work withdrawal, such as absenteeism [26], especially in early childhood education settings. This lack of attention is problematic considering the pervasive nature of teacher absenteeism in low- and middle-income countries.

To address this gap, we employ conservation of resources theory [27] to test whether early childhood teachers’ emotional labor strategies (i.e., surface and deep acting) predict absenteeism. Further, we explore the mediating roles of negative affect and psychological meaningfulness to contribute to the scant literature on teacher emotional labor and absenteeism. In practice, our findings hold education management implications for school headteachers and policy makers in developing policy and program interventions that may reduce workplace absenteeism by working to improve teacher self-regulation, thereby impacting education quality before teachers quit.

## 2 Theoretical and hypotheses development

### 2.1 Ghana context

In attempt to achieve Sustainable Development Goals (SDGs) in education, the Government of Ghana in collaboration with development partners have introduced a number of programs to increase enrolment and achieve quality in early childhood education. These programs include free Compulsory Universal Basic Education in 2007, free school feeding programs, and competency-based curriculum for early childhood education [28]. These developments led to an increased enrolment rate of 74.7 % in 2020 [29]; however, concerns exist about quality standard of education.

In an effort to address these quality problems, the Government of Ghana expanded 2-year early childhood education duration to 3 years by introducing one-year pre-kindergarten in 2018, with hopes of increasing teaching quality and learning outcomes when pupils spend enough time with teachers [30]. Additionally, a 2-h professional learning community program per week has been implemented to help teachers to work in groups and reflect on ideas and practices that can improve teaching and learning process [31]. Despite a variety of intervention efforts, these programs have had little, and at times negative, impact on early childhood education and students’ future learning outcomes [[10], [11], [12]]. For instance, only 28.3 % of fourth grade students attained English Proficiency and 19.8 % attained mathematics accuracy in a national standardized test in 2022 [32]. All these statistics on educational outcomes confirm the conclusion that Ghanaian students show a shallow learning profile [6].

**Fig. 1:**
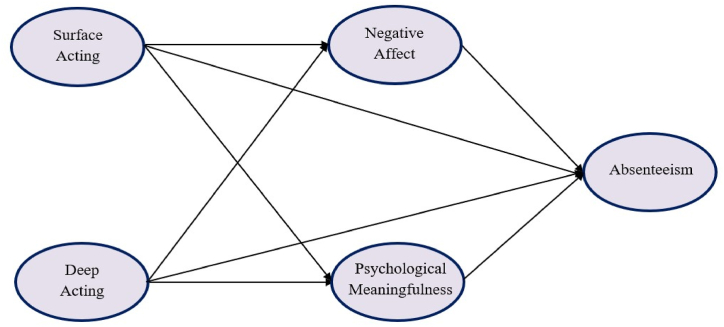
The theoretical model.

Research has documented that the challenges of early childhood education quality are greatly influenced by teachers' negative job behaviors emanating from the complex web of poor work conditions including low salary, late payment, workload, lack of opportunities for professional development, and lack of support [13,33]. These workplace conditions harm preschool teachers' wellbeing [34,35]. Teachers impaired well-being evokes negative job attitudes and behaviors. For example, Peele and Wolf [36] found that Ghanaian preschool teachers' anxiety and depression symptoms predicted lower job motivation and job satisfaction but a higher emotional exhaustion as well as long-term impact on days they were absent from work duties over a school year. Whilst these studies provide understanding of impact of preschool teachers' well-being on absenteeism in low- and middle-income countries, little is known about how meeting schools' emotional display rules impact teachers' absenteeism in Ghana. Considering the degree of success of past and current interventions to get teachers back to the classroom [37], it is important to examine other avenues for effective intervention to improve teachers' attendance to achieve quality preschool education. Therefore, exploring the relationship between teachers’ emotional labor and absenteeism, and the mediation roles of negative affect and psychological meaningfulness is significant for understanding effective approach to provide support and inspire higher teacher attendance rate (see Fig. 1).

### 2.2 The conservation of resources theory

The conservation of resources theory [27] explicates individuals’ motivation to protect existing resource and obtain new ones to facilitate goal achievement [38]. Accordingly, Halbesleben et al. [39] referred to resource as "anything perceived by an individual to help attain his or her goals" (p. 1338). This implies that resource is essential for survival [40]. The conservation of resources theory [38] postulates that circumstances that threaten resource loss and/or cause actual loss at workplace are occupational demands. Research has associated resource loss with psychological, physiological, and behavioral outcomes such as burnout, mental health, satisfaction, negative emotions, sleep disorder and absenteeism [41], enforcing the hypothesis that workplace stressor can induce negative job outcomes.

The conservation of resources theory argues that people are not only docile in coping with stress, but rather expend resource to avert resource loss and achieve their expected goals. However, employees have difficulty balancing the demands of display rules with inadequate resources, leading to strains and then a negative behavioral outcome over time. In low- and middle-income country like Ghana, early childhood teachers lack resources to effectively manage their emotions to reduce strains and may impoverish well-being as well as negative work behavior. Given that emotional labor strategies may deplete or conserve resources, we expect that the emotional labor strategies (surface acting and deep acting) deployed by Ghanaian teachers would trigger either negative affect or psychological meaningfulness, which may then orchestrate or reduce their absenteeism behavior, respectively (see Fig. 1).

### 2.3 The direct relationship between emotional labor and absenteeism

Teaching at the preschool demands not only academic skills but high levels of emotional engagement and management. For preschool teachers to establish genuine and meaningful connections with young children, parents, and colleagues, they are required to manage and redirect emotional situations on a daily basis of which some may be unpleasant, or hostile is seen as emotional labor. Emotional labor refers to management of feelings to create and express a desired facial and bodily display to meet organizational goals [22]. The two main strategies recognized and deployed by employees include surface acting and deep acting [21,22]. Surface acting refers to the act of managing outward expressions by faking unfelt emotion and/or hiding felt emotions whereas deep acting involves managing feelings by changing felt emotions to experience and produce required ones to meet schools' display rules’ demands [25]. It is important to note that, employees deploy surface acting and deep acting as compensatory strategies to spontaneously express desirable feelings to meet organizational goals [42,43].

A good number of contemporary empirical research indicates the distinct consequences of surface and deep acting. For example, meta-analytic findings demonstrate that surface acting deteriorates well-being and job performance and heightens stress and emotional exhaustion [44,45]. Other scholars have demonstrated that surface acting increases negative affect and work withdrawal behavior [46,47] as well as drives negative experiences into individual's home life [48]. The justification for this relationship is that surface acting does not change the felt emotion, which induces emotional dissonance (i.e., discrepancies between felt and expressed emotion) [25]. Again, surface acting requires great effort and costs depletion of limited resources [40,49]. Conversely, deep acting is related positively to job satisfaction, organizational commitment, and job performance [50]. This is because deep acting requires little cognitive effort, it reinstates resources by diminishing the discrepancy between internal feelings and external expressions that may cause resource loss [25].

According to conservation of resources theory, employees are motivated to prevent further resource loss when losing resources. Teachers who constantly engage in surface acting experience resource loss and so they strive to prevent further resource loss by withdrawing from job duties [46,51]. On the other hand, teachers who dominantly engage in deep acting conserve resource, use available resource to promote engagement, self-efficacy, and reduce absenteeism [52]. Therefore, we expect early childhood teachers in Ghana who manage emotions through surface acting to have high absenteeism rate as a strategy of protecting against additional losses. However, Ghanaian early childhood teachers who deep act may feel motivated, committed, and more engaged, thereby reporting less absence from one's work. We thus put forward the following hypotheses:

Hypothesis 1a (H1a): Surface acting relates positively to absenteeism.

H1b Deep acting relates negatively to absenteeism.

### 2.4 The mediating role of negative affect in emotional labor and absenteeism relationship

According to conservation of resources theory [27], people's personal resource repository becomes threatened when expended towards meeting emotional display rules without positive returns, resulting in strains [40]. Gross and John [53] demonstrate that employees experience strain such as negative affect as immediate consequence of stressors emerging from emotional labor. Negative affect is a subjective distress, characterized by a wide range of aversive hostile mood states such as nervousness, anger, contempt, and guilt [54]. These aversive feeling states consume cognitive, emotional, and physical resources to trigger psychological, physiological, and behavioral outcomes. Such link echoes the stressor-strain-outcomes model [55]. Research has identified unpleasant affective states such as anger and anxiety as core factors of teachers' well-being and decision to absent from work duties as well as quitting their profession.

Peele and Wolf [36], studying preschool teachers in Ghana, found lower professional well-being and higher absenteeism over the course of one academic year were induced by strains associated with anxiety and depression symptoms. Teachers who experienced prolonged negative affect were more likely to suffer loss of professional well-being and be absent from school. Zembylas [56] noted a participant in a qualitative study saying s/he “feeling overwhelmed, frustrated or angry” was an example of teachers feeling aversive emotions which are often harmful emotionally and professionally. Because it takes a lot of energy to deal with frustration. And it affects teachers' performance in the classroom. The causes of negative affect as suggested by emotion regulation process model are suppression (i.e., surface acting) and reappraisal (i.e., deep acting). These emotion regulation strategies play critical role in individuals’ affective states [53]. Specifically, surface acting heightens negative affect while deep acting reduces negative affect, and the subsequent implications for work withdrawal behavior [46].

We argue that performing surface acting may induce negative affective state (such as fear, disgust, guilt, and distress) and fatigue due to inauthenticity [57]. It is important to note that, being inauthentic or expressing insincere emotion at workplace is considered as lying [58]. So then, surface acting is seen as lying to oneself and others. For example, a participant recounted in Yin and Lee [58] that “even if you are in pain, you have to smile to the students, give students a smile when you stand up on the speaker's platform” (p. 61). Yin and Lee [58] posited that teachers surface act for variety of reasons: to achieve teaching goals, maintain good relationship with students, co-workers, and parents, and protect their image. Research demonstrates that surface acting has detrimental psychological and physiological consequences. A study of student nurses found traces of muscular actions associated with disgust, fear, contempt, and sadness are as results of faking to conceal negative emotions [59]. Similarly, Tomura [60] found that faking or hiding one's true identity is associated with stress, anxiety, and exhaustion. Conversely, repeated deep acting would create desired emotions that commensurate with display rules in a job setting, promote positive job attitudes and behaviors. Because any emotion displayed using deep acting is genuine or truly felt emotion, leading to authenticity [40]. Research demonstrates that deep acting decreases negative affect and increases positive affect [46]. This positive emotion nurtures employees' well-being, performance, and satisfaction as well as reduces emotional exhaustion and work withdrawal [61].

To sum up, teachers’ emotional strategies may predict teacher absenteeism indirectly through negative affect. Particularly, teachers who lie about their emotions through surface acting heightens their negative affect because of extensive tension causing resource depletion, which in turn, lead to work withdrawal [46,47]. However, teachers who sincerely express positive emotions through deep acting conserve personal resource to enhance authenticity, sense of accomplishment, and reduce negative affect, which in turn predict work withdrawal. Therefore, we hypothesize the following:

H2 *Negative affect mediates the relation between (a) surface acting and (b) deep acting and absenteeism.*

### 2.5 The mediating role of psychological meaningfulness in emotional labor and absenteeism relationship

The concept of psychological meaningfulness refers to the level of importance and value individuals perceive their work situation [62]. Psychological meaningfulness plays an essential role in individuals' well-being and life satisfaction. Perceiving meaning and purpose in our lives through work roles reflect individual sense of coherence, direction, and fulfilment in life [63,64]. And this is driven by the alignment of personal beliefs, values, and goals with one's work actions and experiences. Employees who identify their work to be personally meaningful perceive their contributions as valuable, significant, and fulfilling, which may lead to greater life satisfaction, job satisfaction, organizational commitment, and organizational citizenship behavior, as well as low absenteeism and turnover [64,65].

However, perceiving a sense of meaning at workplace can be somewhat challenging. This is especially true for people-service employees who are notably known for engaging in emotional labor [21,22]. According to the conservation of resources theory, performing emotional labor (i.e., surface acting or deep acting) can threaten or enhance the decency and meaningfulness of work because of resource loss or gain tendencies [66]. In particular, engaging in surface acting may constitute a distraction for individuals psychology meaningfulness. That is, the emotional dissonance individuals undergo when engaged in surface acting may be seen as a challenge for identifying the essential part of their job [25]. Research indicates that surface acting is associated with low psychological meaningfulness [67]. This lack of meaning in work may cause employees to alienate or disengage from their work [68].

In contrast, deep acting has been associated with individuals' committed effort towards feeling the desired emotion and being authentically themselves. The ability to change felt emotions to align with expressed emotions may evoke teachers’ sense of purpose and personal identity as educators, because their emotions align with their values and beliefs [50,69]. For instance, Sutton [70] found that teachers who believed that managing emotions enhanced their teaching effectiveness and conformed to their idealized emotion image of a teacher were less vulnerable to burnout. In fact, teachers who were successful in meeting emotional demands were those who deep acted. They found their work meaningful by fitting well into their roles and perceiving their interaction with students, co-workers, and parents as authentic and emotionally meaningful [68]. Teachers who meet display rules through sincere emotional expressions make their workplace more comfortable where they can access support, meaning, peace, and personal fulfilment [50,69]. Similarly, teachers with high sense of meaningfulness feel happy, content, and fulfilled with their work, and develop harmonious passion [71]. They engage in variety of instructional strategies, manage classroom behaviors, motivate and help students to learn and achieve success [72]. They also develop a sense of professional commitment, engagement, and high performance [73], and reduced work withdrawal behaviors [74].

Consistent with the above, it is plausible to argue that teachers who surface act may find work less meaningful whereas teacher who deep act may find work more meaningful, develop identity, and self-actualize, which in turn influence the motivation to not be absent from work duties [75]. Considering the relationship between emotional labor (surface and deep acting) and psychological meaningfulness and absenteeism, we therefore hypothesize that:

H3 *Psychological meaningfulness mediates the relation between (a) surface acting and (b) deep acting and absenteeism.*

## 3 Method

### 3.1 Participant and procedure

We obtained approval (No: ZSTR2023152) from Zhejiang Normal University's Ethics Review Committee and Ghana Education Service to conduct this study. Participants were early childhood teachers from public and private schools in Kwabre East and Atwima-Nwabiagya districts from Ashanti region of Ghana, where teaching and caring for pupils are the core components of their duties [76]. The districts host a variety of activities that showcase the diverse ethnic, cultural, technology, economic, and administrative mix and therefore enlist teachers and pupils with varied techno-economic and socio-cultural backgrounds. All schools totaling 365 were identified from the two district offices, 115 constituted public schools and 250 were private schools. Given the support from Ghana Education Service, all the public schools within these districts had to participate in the study, but private schools were not mandated therefore more refused to participate in the study. Ninety-five (95) public and 195 private schools were contacted, and 82 private schools agreed to participate in the study. A final sample of 177 schools of which 95 were public and 82 were private schools were used for the study.

Most of the schools had two preschool classes (i.e., KG1 comprising of four years and KG2 five years old pupils) with one to five teachers, and embrace inclusive education, where students with and without special developmental needs are listed together in the same class. At most, four preschool teachers (two each from KG1 and KG2) were sampled per school and randomly selected if they were more than four preschool teachers. The data were collected after the informed consents of the teachers. All data were collected through a survey administration except absenteeism data, which were taken from the attendance records of each school. Upon completion of the survey, teachers were given souvenirs as a sign of appreciation. A final sample of 574 early childhood teachers, consisting of 377(65.7 %) female and 197(34.3 %) male teachers, with majority having diploma or higher certificates 461(80.4 %), and are public preschool teachers 357(62.2 %). Their ages, work experiences and class sizes range from 18 to 59 years (Mean = 32.21, SD = 8.07), 5–37 years (Mean = 8.45, SD = 5.66), and 9–60 pupils (Mean = 36.30, SD = 13.18), respectively. Please see web appendix Table 1 for more details.

### 3.2 Measures

***Emotional labor.*** Teachers’ emotional labor (i.e., surface and deep acting) were measured with 10 items from Teacher Emotional Labor Strategy Scale (TELSS) adapted by Yin [77]. Surface acting comprises of six items (e.g., I fake the emotions I show when dealing with students or their parents), and deep acting consists of four items (e.g., I work at developing the feelings inside of me that I need to show to students or their parents). Teachers indicated their agreement with the item on the scale from (1-strongly disagree to 5-strongly agree). Our study revealed the sub-scales had internal consistency at α = .88 and α = .81 for surface acting and deep acting, respectively.

***Negative affect*.** Negative affect was measured using PANAS developed by Watson et al. [54]. The negative affect scale consists of 10 items (e.g., Upset, Nervous, etc.). Teachers related the items to their mood at workplace and rated them on a 5-point scale ranging from (1-Not at all to 5- extremely). The scale had a very good internal consistency at α = .98.

***Psychological meaningfulness.*** Psychological Meaningfulness was measured with six items (e.g., The work I do on this job is very important to me) compiled by May et al. [64]. This scale assesses the degree of meaning that individuals perceive in their job-related activities. Teachers responded to each item on a 5-point Likert scale ranging from (1-Strongly disagree to 5-Strongly agree). The internal consistency obtained for this scale was α = .88.

***Absenteeism*.** Absenteeism data were sourced from each school's administrative record book (i.e., logbook). Absenteeism was measured as the total number of days teachers were absent from work duties in an academic year (Mean = 24.05, min = 13, max = 35). Absent days include sick leave and voluntary absences. However, vacation, public holiday, and maternity leave as well as partial absences such as late arrivals or early departures were not considered as absent days in this present study. Although the total number of school days vary between private and public kindergartens (i.e., two weeks more than the public schools' in a year), the private schools have 9–12 days paid leave in a school year known as mid-term holidays, making school days approximately equal for both private and public kindergartens. Since our data was collected in the third term (i.e., last term of the academic year), we had the chance to accumulate the absent days from term 1 to term 3 for each participant. Hammer et al. [78] suggested that using one calendar year data is sufficient and allow a reliable number of incidences because of possible low absenteeism rate within short time.

***Covariates.*** As age, gender, educational level, school type, work experience, and class size have been found to account for the differences in negative emotions, psychological meaningfulness, and absenteeism [22,46], these variables were also included in our model as control variables for the possibility of spurious relations among the study variables. These variables were measured as follows: gender (0 = Male & 1 = Female), age (teachers were asked to state their age), education level (0 = High school and lower, 1 = Diploma/Higher National Diploma & 2 = bachelor degree and above), school type (0 = public school & 1 = private school), work experience (teachers were asked to state their work experience in years), and class size (teachers were asked to state their class size). See web appendix Table 1 for detailed participants’ information.

### 3.3 Statistical procedure

To ensure the quality of the data, several analytical procedures were conducted. First, we examined missing data patterns using Little's “missing completely at random” (MCAR) test (χ^2^ = 232.386, df = 246, p = .740) and subsequently, treated the missing data with full information maximum likelihood [79]. Also, the presence of common method variance (CMV) [80] was assessed using Harman's one factor analysis. The result accounted for 36.19 % of the total variance, indicating no serious common method bias issue. Further, due to the potential hierarchical nature of the data (i.e., teachers were nested in schools), the intraclass correlation coefficient (ICC) was performed to examine the proportion of variance in each dependent variable occurring at the higher level of analysis (i.e., school-level). The results of ICC for each outcome variable showed a small proportion of variance explained by the school level (see Table 2), meaning utilizing normal regression model is appropriate [81].

Second, descriptive statistics, inter-constructs correlation, and measurement model utilizing confirmatory factor analysis (CFA) to determine the uniqueness of the latent factors were conducted. To evaluate the models' fit, Hu and Bentler's [82] Chi-Square statistics (χ^2^), the Root Mean Square Error of Approximation (RMSEA ≤.08), Comparative Fit Index (CFI ≥.90), Tucker–Lewis Index (TLI ≥.90), and Standardized Root Mean Square Residual (SRMR ≤.08) were used for acceptable fit for both measurement and structural models. Last, to test the study hypotheses, structural equation model (SEM) was conducted to examine the various relationships between the constructs of interest. SEM can estimate all effects and integrate them into one complex model, account for measurement errors, and give accurate and stable results for studied constructs [83]. All analyses were conducted with Mplus version 8.3 using maximum likelihood estimation [84].

## 4 Results

### 4.1 Measurement model

The measurement model showed a goodness fit of our hypothesized model to the data (χ^2^ = 490.25, df = 236, χ^2^/df = 1.77, CFI = .97, TLI = .97, RMSEA = .05, SRMR = .03), and was better than the alternatives after series of item-level modifications (see Table 1).

**Table 1:** Fit indices for measurement models.

Model	χ^2^	df	CFI	TLI	RMSEA	SRMR	SCF	Δχ^2^_SB_	Δdf
1. Four-factor model	490.25	236	.97	.97	.05	.03	1.02		–
2. Three-factor model (SA-DA = 1 factor)	1055.94	239	.92	.90	.08	.07	1.04	228.90∗∗∗	3
3. Three-factor model (NA – Meaning = 1 factor)	1507.91	239	.87	.85	.10	.11	1.05	317.67∗∗∗	3
4. Two-factor model (SA-DA = 1 factor and NA – Meaning = 1 factor)	2061.93	241	.81	.79	.13	.13	1.07	497.44∗∗∗	5
5. Two-factor model (SA-DA-NA–Meaning = 1 factor)	2814.54	242	.74	.70	.15	.15	1.10	610.81∗∗∗	6

Note: N = 574, CFI = comparative fit index, TLI = Tucker–Lewis index, RMSEA = root mean square error of approximation, SRMR = standardized root mean square residual, SCF = scaling correction factor, Δχ^2^_SB_ = strictly positive Satorra-Bentler chi-square difference test, χ^2^ = chi-square, df = degree of freedom, Δdf = difference in degree of freedom, DA = deep acting, SA = surface acting, NA = negative affect, Meaning = psychological meaningfulness, Absent = absenteeism. ∗∗∗*p* < .001.

**Table 2:** Descriptive statistics, reliability and validity, ICC, and partial correlation among constructs.

	CR(α)	AVE	MSV	MaxR(H)	1	2	3	4	5	6	7	8	9	10	11
1. Age					–										
2. Sex					−.02	–									
3. Education level					.21∗∗∗	−.05	–								
4. School type					−.04	−.08	−.49	–							
5. Work experience					.24∗∗∗	.08	−.02	−.02	–						
6. Class size					.12∗∗	−.00	.14	−.08	.08	–					
7. Absenteeism					.02	.07	.03	−.04	.02	−.03	–				
8. Negative affect	.98(.98)	.80	.17	.98	−.06	.08	−.08	.03	.05	−.07	.32∗∗∗	.894			
9. Psychological meaningfulness	.88(.88)	.55	.17	.89	−.02	−.05	.04	.02	−.04	.14∗∗	−.30∗∗∗	−.41∗∗∗	.743		
10. Surface acting	.87(.88)	.62	.16	.93	−.01	.04	−.05	−.01	−.05	−.05	.37∗∗∗	.41∗∗∗	−.38∗∗∗	.790	
11. Deep acting	.81(.81)	.52	.08	.82	−.00	−.04	.08	−.04	.06	.17∗∗∗	−.17∗∗∗	−.14∗∗	.28∗∗∗	−.26∗∗∗	.723
Mean					32.21	1.66	2.33	1.38	8.45	36.30	24.05	1.95	3.61	2.10	3.68
SD					7.24	.48	.79	.49	5.67	13.18	2.81	1.06	1.02	.77	1.06
ICC											.037	.010	.020	.050	.085

*Note*: SD = standard deviation, ICC= Interclass correlation, CR = composite reliability; α = Cronbach's alpha; AVE = average variance extracted; MSV = maximum shared variance; MaxR(H) = maximal reliability, diagonal values are square root of average variance extracted, values in columns numbered 1–11 are inter-constructs correlation.

∗*p* < .050, ∗∗*p* < .010, and ∗∗∗*p* < .001.

The constructs' discriminant validity was also examined. In line with previous studies [85,86], the average variance extracted (AVE) (.52 ≤ AVEs ≤.80) were greater than mean shared variance (MSV) (.08 ≤ MSVs ≤.17), and the square root of AVE (√AVE) (.72 ≤ √AVEs ≤.89) were also greater than inter-construct partial correlation (−.36 ≤ *r* ≤ .41) [87]. In addition, convergent validity was met with AVEs being greater than .50. Again, constructs' internal reliabilities (.81 ≤ *a* ≤ .98), composite reliabilities (.81 ≤ *CR* ≤ .98), and maximal reliability (.82 ≤ *a* ≤ .98) met the minimum threshold (70). The constructs' descriptive statistics (mean ± SD), validity and reliability, AVE, MSV, √AVE, composite reliability, Cronbach's alphas, maximum reliability, and inter-constructs’ correlation coefficients have been presented in Table 2.

### 4.2 Hypotheses test

**Fig. 2:**
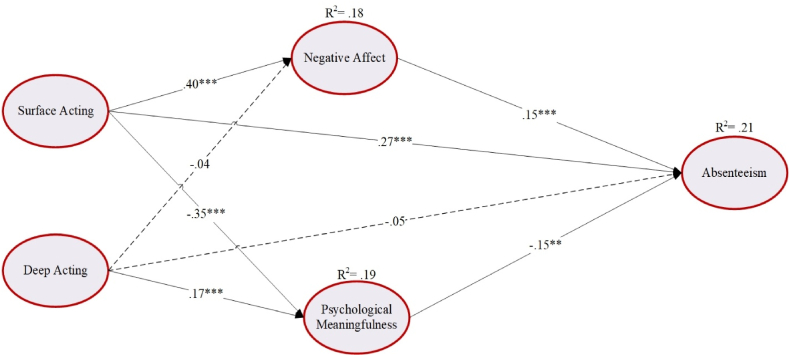
Results of the direct relations between emotional labor, negative affect, psychological meaningfulness, and absenteeism. Note: The solid lines represent significant paths and dashed lines are nonsignificant relationships, the values are standardized estimates. ∗*p* < .050, ∗∗*p* < .010, and ∗∗∗*p* < .001.

Using SEM, the mediation model was estimated to explore the relationship between emotional labor (i.e., surface acting and deep acting) and absenteeism through negative affect and psychological meaningfulness, controlling for the probable confounding effect of teachers’ demographic profiles (i.e., gender, school type, and education level were dummy coded, and age, work experience, and class size were treated as continuous variables) in the model. A biased-corrected bootstrapped method with 10,000 bootstrap samples and 95 % confidence interval (CI) on our lantent constructs was used. The model provided a close fit to the data (χ^2^ = 757.77, df = 411, χ^2^/df = 1.84, CFI = .97, TLI = .97, RMSEA = .04, SRMR = .06). Fig. 2 presents the standardized estimates of the direct relationships. In line with H1a, H2a and H3a, the results revealed that surface acting had a significant positive association with absenteeism (β = .27, *SE* = .05, p < .001) and negative affect (β = .40, *SE* = .05, p < .001), but negative with psychological meaningfulness (β = −.35, *SE* = .05, p < .001). Deep acting significantly related positively to psychological meaningfulness (β = .17, *SE* = .05, p < .01) supporting H3b. Again, negative affect significantly predicted absenteeism (β = .15, *SE* = .04, p < .001), and psychological meaningfulness had significant negative association with absenteeism (β = −.15, *SE* = .05, p < .01) are in line with H2 and H3 (see Fig. 2 & web appendix Table 2).

Last, the results of indirect relationship between emotional labor (i.e., surface acting and deep acting) and absenteeism through negative affect and psychological meaningfulness revealed a significant association between surface acting and absenteeism (β = .06, 95 % CI [.039, .100]; β = .05, 95 % CI [.018, .091]) via negative affect and psychological meaningfulness respectively supporting H2a & H3a. Again, deep acting had significant indirect association with absenteeism (β = −.03, 95 % CI [-.054, −.008]) through psychological meaningfulness supporting H3b. Because the direct association between surface acting and absenteeism was significant, we henceforth established that negative affect and psychological meaningfulness partially mediate the relationship between surface acting and absenteeism, but psychological meaningfulness fully mediates the deep acting and absenteeism relationship (see Table 3).

**Table 3:** Indirect effect of emotional labor on teacher absenteeism, via negative affect and psychological meaningfulness.

Indirect Path	Standardized Estimate	SE	95 % BCCI		P-Value
			Lower	Upper	
SA → NA → Absent	.061∗∗	.018	.029	.100	.001
SA → Meaning → Absent	.051∗∗	.018	.018	.091	.006
DA → NA → Absent	−.006	.007	−.024	.006	.402
DA → Meaning → Absent	−.025∗	.011	−.054	−.008	.022

Note: DA = deep acting, SA = surface acting, NA = negative affect, Meaning = psychological meaningfulness, Absent = absenteeism, SE = standard error, CI = confidence interval.

∗*p* < .050, ∗∗*p* < .010, and ∗∗∗*p* < .001.

## 5 Discussion

Many low- and middle-income countries such as Ghana have managed to increase the number of children not up to school-going age in kindergartens. However, the enrolment increases have only compounded quality concerns [88]. A number of factors including preschool teachers’ professional development and motivation have been examined from structural quality perspective [9,12,13,89]. We argue that these structural quality challenges including teacher-student ratio, lack of teaching and learning materials may lead to a critical job withdrawal behavior which is pervasive in low- and middle-income countries – teacher absenteeism. The literature evidence is clear on the deleterious effects of teacher absenteeism on education quality [19,36]. However, the scant studies on teacher absenteeism have explored only the distal factors of how classroom structural quality induces teacher absenteeism. We advance this research by examining the proximal elements that are the immediate cause of teacher absenteeism. Particularly, the present study examined the association between emotional labor (surface acting and deep acting) and absenteeism among early childhood teachers in Ghana, and the mediating effects of negative affect and psychological meaningfulness using conservation of resources theory by Hobfoll [27] to explain the underlying mechanisms.

As hypothesized, the findings of our study suggest that surface acting relates positively to absenteeism. Moreover, both negative affect and psychological meaningfulness mediated the association between surface acting and absenteeism. Finally, psychological meaningfulness mediated the association between deep acting and absenteeism.

### 5.1 Theoretical and practical implications

Our research makes insightful theoretical contributions to the early childhood teachers' literature in two ways. First, we add to the research on the antecedents of absenteeism among teachers who face poor working conditions such as low pay, high emotional demands, and lack of opportunities for professional development [37]. Specifically, surface acting was found to have a positive association with absenteeism. This finding is consistent with the conservation of resources theory [27] and other empirical research conducted among people-service professionals that surface act. For instance, Yin et al. [45], through meta-analysis, revealed the deteriorating effects of teachers' surface acting on employees' well-being and organizational outcomes. This echoes the broad literature on occupational stress [90]. That is, it supports the research evidence that meeting emotional display rules through surface acting costs individuals' well-being and motivation to attend and engage in work duties [21]. Again, from a conservation of resources perspective, the results confirm Hobfoll's protection mechanism; that is, when valued resources are threatened or lost as a result of surface acting, people will strive to protect and/or prevent further resource loss by withdrawing from work roles that are capable of depleting resources [27]. It is worth noting that, absenteeism is not the best way to curb the effect of resource loss at workplace, but somewhat aids in the process of replenishing expended resources [26]. Future research could design an interventional study aimed at modifying teacher behavior and enhancing resources as necessary levers to improve educational quality, especially in low- and middle-income countries.

Second, the findings reveal that, distinctly from one another, both negative affect and psychological meaningfulness partially mediated surface acting and absenteeism link. Regarding the mediating role of negative affect, the results are in sync with prior findings that demonstrate that negative affect positively relates to employees' ill-being [44,54]. That is, early childhood teachers who surface act by feigning emotion heighten their negative feelings as a result of emotional dissonance experience, which in turn lead to work withdrawal such as unplanned time off from work [46]. Also, the mediating role of psychological meaningfulness in surface acting and absenteeism link was established. This finding supports the general literature that stress harms well-being [91] by sapping motivational resources [38,92] that enable productive behavioral outcomes [68]. Moreover, concerning the mediating role of psychological meaningfulness in deep acting and absenteeism relationship, this finding supports prior research demonstrating that individual psychological meaningfulness at workplace nurtures commitment, life satisfaction, and work engagement [64,73]. These findings indicate that, whichever emotional labor strategy teachers employ to meet display rules plays a role in one's role fit or conflict [44]. In educational setting, meaningfulness has been seen as subjective [63,93]. However, the literature in the field of political theory suggests that meaningfulness constitutes not just subjective but also objective constructs, that is, work that promotes autonomy, dignity, freedom, security, and safety [94]. Future research could assess the objective dimensions of meaningfulness of work within teaching profession and link to job outcomes associated with quality education.

The present study has a number of practical implications for policy makers, school administrators, and headteachers. First, our findings revealed that meeting emotional labor demands is not always associated with negative work outcomes. Such negative outcomes do only emerge when teachers engage in surface acting as a preferred strategy, which is a potential threat to resource loss. Conversely, deep acting is clearly not detrimental but rather nurtures positive job outcomes. Our research findings emphasize the need for policy makers to restructure emotional demands placed on employees and design training programs in cohorts that will enhance teachers emotional labor strategies and skills development. Preschool education management should organize emotional labor training that can promote awareness on the different types of emotional labor strategies, and its advantages and disadvantages, in order to lessen emotional susceptibilities among teachers [95]. Also, management and headteachers should make effort to create a work environment that would foster and value authentic emotional expression (i.e., deep acting) because deep acting consumes less resource and aids in resource protection against loss. Such approach would address emotional dissonance resulting from surface acting through supportive policies and training programs. These training programs can be in the form of seminars, workshops, talk shows, and coaching that would educate teachers on emotional demands and empower them to feel greater sense of ownership when enacting emotional labor strategies in and outside classroom interactions.

Again, school administrators and headteachers should clearly communicate educational goals, values, and mission to teachers as well as their job roles and responsibilities in order to avoid ambiguity of teachers' contributions. Policy makers and management should redesign teachers' roles to ensure autonomy and offer opportunities for professional development. That is, allow autonomy at workplace and provide opportunities for teachers to learn new skills and grow within the teaching profession. This will enhance their sense of actualization and meaningfulness [96]. Educational management should establish a positive culture that acknowledges and rewards teachers' contributions. When teachers feel valued and appreciated at the workplace, their sense of meaningfulness increases and reduces the desire to be absent from work duties. Again, psychological meaningfulness of work features should be periodically assessed on employees' surveys. Items should consider individual's levels of self-actualizing work, realization of purpose, goals and values, feelings of personal accomplishment, and perceived ability to meet one's highest career goals within the teaching profession.

Finally, educational management and policy makers should introduce school- and teacher-level interventions that would help reduce absenteeism among teachers. This intervention includes adaptation of a management style that is responsive to teachers’ needs. That is, involve teachers in the development of pay incentive plans [97]. Again, management and headteachers should introduce behavior modification control programs that will help to increase punctuality and decrease absenteeism [98]. This program can be monitoring and posting attendance figures, designing posters, creating competition between teachers, rewarding attendance and penalizing absenteeism related behaviors to reduce absenteeism [37].

### 5.2 Limitations and future research directions

Though the present study offers significant contributions, several limitations should be acknowledged. First, testing our theoretical model with self-reported data might have influenced the relationships observed [80]. However, the sensitivity of the data to common method variance revealed no issue to our findings after a number of statistical and procedural measures, including: (a) attention check item to check to ensure the quality of responses to the questionnaire and (b) Harman's single-factor EFA statistical technique was used to rule our common method bias. Also, the method used prevents us from examining teachers' use of surface acting and deep acting in the context of particular emotional episodes. We recommend that future research should use diary or experience sampling method to assess emotional labor strategies employed by teachers and map them to specific emotional experiences and behavioral outcomes. Last, given that the data for this study is from low- and middle-income country, it may be plausible to apply the findings in countries with similar working conditions. However, such application must be done cautiously, taking into account the study's inherent limitations. Ideally, it would be best to replicate this study in other low- and middle-income countries.

### 5.3 Conclusion

In conclusion, our study sought to extend the literature on teacher absenteeism by examining the proximal factors rather than the usual distal factors such as work conditions, teacher motivation, and professional development. To achieve our objective, we sampled preschool teachers from kindergartens in two districts of Ashanti region of Ghana. Our findings provide richer understanding of how the proximal factor of emotional labor strategies relate to teacher absenteeism among early childhood teachers in Ghana. Specifically, the results of this research demonstrate that surface acting promotes absenteeism. Also, negative affect and psychological meaningfulness mediated the relationship between surface acting and absenteeism. We discuss these findings in the context of our theory and extant empirical research and proffer a number of practical recommendations to help reduce preschool teacher absenteeism, thereby improving preschool education quality in Ghana.

## CRediT authorship contribution statement

**Seth Yeboah Ntim:** Writing – original draft, Methodology, Funding acquisition, Formal analysis, Conceptualization. **Collins Opoku Antwi:** Writing – review & editing, Writing – original draft, Supervision, Conceptualization. **Michael Osei Aboagye:** Resources, Project administration, Formal analysis, Data curation. **Elijah Takyi Mensah:** Writing – review & editing, Writing – original draft, Methodology. **Emmanuel Tetteh Teye:** Writing – review & editing, Methodology, Formal analysis, Data curation. **Xinyu Li:** Supervision, Project administration, Conceptualization.

## Data availability

Data will be provided upon request.

## Funding

This work is funded by Zhejiang Normal University Postdoctoral Research Fund, China [YS304123954].

## Declaration of Competing Interest

The authors declare that they have no known competing financial interests or personal relationships that could have appeared to influence the work reported in this paper.
