# Supplementary material for: Emotional labor and absenteeism among early childhood educators: The mediating roles of negative affect and psychological meaningfulness
Source: Heliyon. 2024 Nov 1;10(21):e40039. doi: 10.1016/j.heliyon.2024.e40039 (PMC11570294; doi:10.1016/j.heliyon.2024.e40039)
Supplement: Multimedia component 1 [file mmc1.docx]

**Appendix**

Appendix: Table 1: Participants personal and job-related characteristics (N=574)

| Variable | | Frequency(%) | Mean±SD |
| --- | --- | --- | --- |
| Sex | |  |  |
|  | Male | 197(34.3%) |  |
|  | Female | 377 (65.7%) |  |
| Age | |  | 32.21±7.25 |
|  | 29 and below | 230(40.0%) |  |
|  | 30 – 39 | 238(41.5%) |  |
|  | 40 and above | 106(18.5%) |  |
| Educational Level | |  |  |
|  | Senior high school and lower | 113(19.6%) |  |
|  | Diploma/Higher National Diploma | 158(27.5%) |  |
|  | Bachelor degree & above | 303(52.9%) |  |
| School Type | |  |  |
|  | Public school | 357(62.2%) |  |
|  | Private school | 217(37.8%) |  |
| Work experience | |  | 8.45±5.67 |
|  | ≤5 years | 203(35.4%) |  |
|  | 6-10 years | 194(33.8%) |  |
|  | ≥11 years | 177(30.8%) |  |
| Class size | |  | 36.30±13.18 |
|  | ≤15 pupils | 42(7.3%) |  |
|  | 16 – 30 pupils | 133(23.2%) |  |
|  | 31 – 45 pupils | 204(35.5%) |  |
|  | ≥46 pupils | 195(34.0%) |  |

Note: SD= standard deviation

Appendix: Table 2: The results of direct relations between emotional labor, negative affect, psychological meaningfulness, and absenteeism

|  |  | Standardized Estimates (SE) | | | | |
| --- | --- | --- | --- | --- | --- | --- |
| Variable |  | Negative affect |  | Psychological meaningfulness |  | Absenteeism |
| Sex (ref: Male) |  | .06(.04) |  | -.03(.04) |  | .04(.04) |
| Age |  | -.09(.12) |  | -.12(.14) |  | -.06(.15) |
| Education level (ref: SHS & lower) |  |  |  |  |  |  |
| Diploma/Higher National Diploma |  | -.01(.06) |  | .00(.06) |  | .06(.06) |
| Bachelor’s degree |  | -.02(.06) |  | .04(.07) |  | .07(.07) |
| School type (ref: Public) |  | .02(.05) |  | .03(.05) |  | -.04(.05) |
| Work experience |  | .05(.13) |  | .09(.14) |  | .02(.14) |
| Class size |  | .04(.04) |  | .10*(.04) |  | -.02(.04) |
| Surface acting |  | .40***(.05) |  | -.35***(.05) |  | .26***(.06) |
| Deep acting |  | -.04(.05) |  | .17**(.05) |  | -.05(.05) |
| Negative affect |  |  |  |  |  | .15***(.04) |
| Psychological meaningfulness |  |  |  |  |  | -.15**(.05) |
| R^2^ |  | .18 |  | .19 |  | .21 |

Note: SE= standard error, SHS= senior high school.

**p* < .050, ***p* < .010, and ****p* < .001


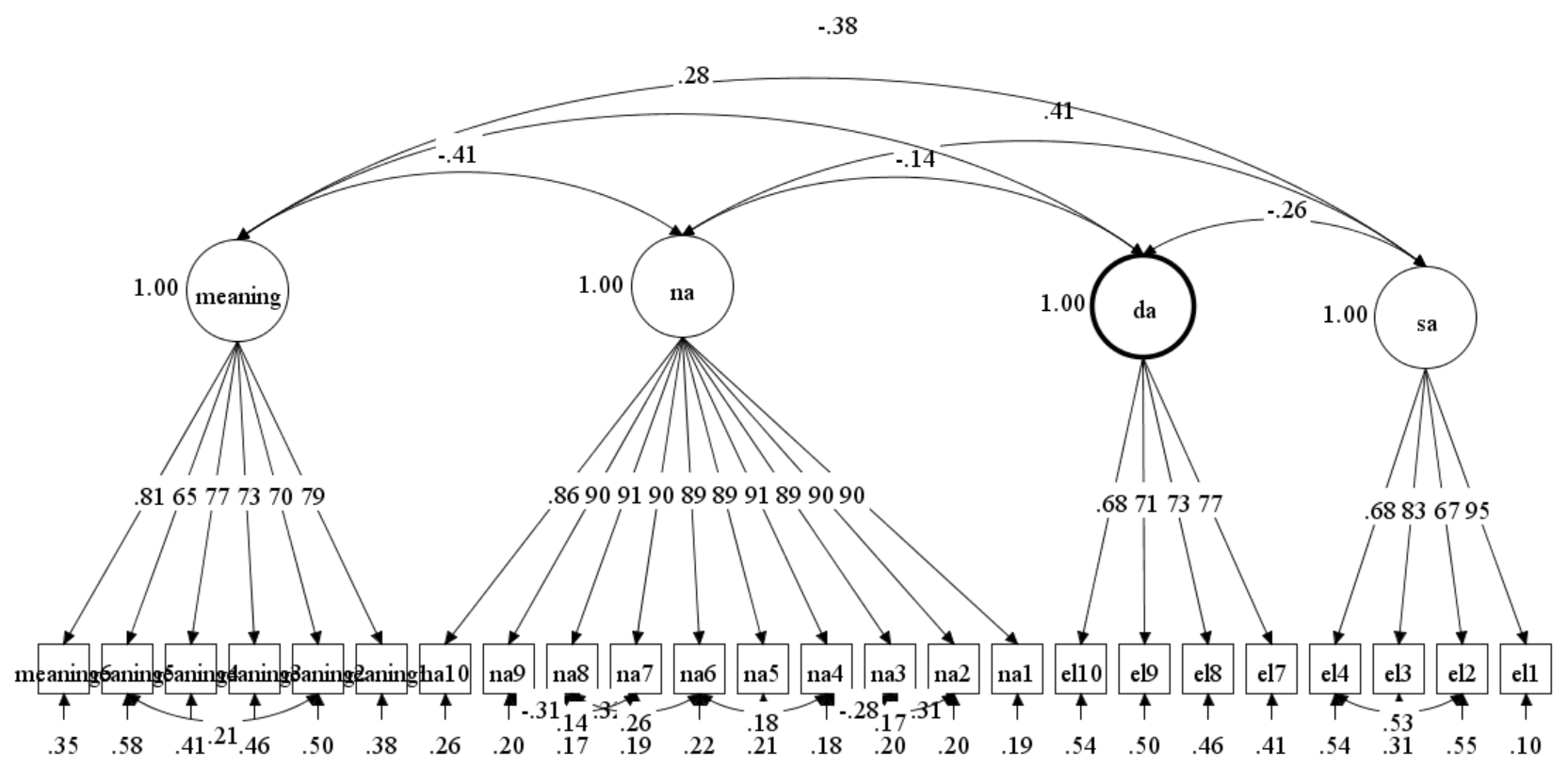


Appendix: Figure 1: Measurement model

Note: meaning = psychological meaningfulness, na = negative affect, da = deep acting, sa = surface acting.

Appendix: Table 3: Items’ descriptive statistics and standardized CFA factor loadings

|  | Normality and descriptive statistics | | |  |
| --- | --- | --- | --- | --- |
|  | Mean±SD | Skewness | Kurtosis | CFA loadings |
| **Surface acting** (Yin, 2012) |  |  |  |  |
| I put on an act in order to deal with students or their parents in an appropriate way. | 2.10±1.04 | 1.02 | .59 | .95 |
| I put on a ‘show’ or ‘performance’ when interacting with students or their parents. | 2.02±1.00 | 1.05 | .83 | .66 |
| I show feelings to students or their parents that are different from what I feel inside. | 2.10±1.08 | .09 | .06 | .86 |
| I fake the emotions I show when dealing with students or their parents. | 2.09±1.04 | .94 | .42 | .67 |
| I just pretend to have the emotions I need to display for my job. | 2.16±1.23 | .96 | -.03 | ….. |
| I put on a ‘mask’ in order to display the emotions I need for the job. | 2.13±1.18 | .85 | -.24 | ….. |
| **Deep acting** (Yin, 2012) |  |  |  |  |
| I try to actually experience the emotions that I must show to students or their parents. | 3.61±1.38 | -.64 | -.90 | .78 |
| I make an effort to actually feel the emotions that I need to display towards students or their parents. | 3.68±1.33 | -.70 | -.70 | .72 |
| I work hard to feel the emotions that I need to show to students or their parents. | 3.52±1.39 | -.58 | -.97 | .70 |
| I work at developing the feelings inside of me that I need to show to students or their parents. | 3.91±1.18 | -.93 | -.07 | .67 |
| **Negative affect** (Watson et al., 1988) |  |  |  |  |
| Afraid | 2.01±1.29 | 1.19 | .18 | .90 |
| Nervous | 1.99±1.29 | 1.22 | .25 | .90 |
| Scared | 1.98±1.31 | 1.26 | .46 | .88 |
| Upset | 1.98±1.31 | 1.25 | .28 | .91 |
| Guilty | 1.98±1.28 | 1.23 | .31 | .89 |
| Hostile | 2.00±1.30 | 1.22 | .25 | .88 |
| Ashamed | 2.04±1.27 | 1.12 | .11 | .91 |
| Jittery | 2.04±1.28 | 1.14 | .13 | .91 |
| Irritable | 2.00±1.27 | 1.16 | .14 | .90 |
| Distressed | 2.01±1.33 | 1.19 | .11 | .86 |
| **Psychological meaningfulness** (May et al., 2004) |  |  |  |  |
| The work I do on this job is very important to me. | 3.65±1.28 | -.56 | -.91 | .79 |
| My job activities are personally meaningfulness to me. | 3.62±1.26 | -.43 | -1.02 | .70 |
| The work I do on this job is worthwhile. | 3.58±1.28 | -.43 | -.94 | .72 |
| My job activities are significant to me. | 3.65±1.33 | -.55 | -.96 | .76 |
| The work I do on this job is meaningful to me. | 3.59±1.26 | -.36 | -1.11 | .65 |
| I feel that the work I do on my job is valuable. | 3.60±1.29 | -.52 | -.90 | .80 |

Note: SD= standard deviation, CFA= confirmatory factor analysis.
